# Major Causes of Perinatal and Paediatric Mortality in Sub‐Saharan Africa and South Asia: Adjustment for Selection Bias in the CHAMPS Network

**DOI:** 10.1111/ppe.70067

**Published:** 2025-09-04

**Authors:** Kartavya J. Vyas, Jonathan A. Muir, Zachary J. Madewell, Priya M. Gupta, Dianna M. Blau, Shams E. Arifeen, Emily S. Gurley, Atique I. Chowdhury, Kazi M. Islam, Afruna Rahman, J. Anthony G. Scott, Nega Assefa, Lola Madrid, Yohanis A. Asefa, Yasir Y. Abdullahi, Dickens Onyango, Victor Akelo, Beth A. Tippett‐Barr, George Aol, Samba O. Sow, Karen L. Kotloff, Milagritos D. Tapia, Adama M. Keita, Kiranpreet Chawla, Quique Bassat, Inacio Mandomando, Ariel Nhacolo, Charfudin Sacoor, Ikechukwu Ogbuanu, Dickens Kowuor, Babatunde Duduyemi, Andrew Moseray, James S. Squire, Shabir Madhi, Sana Mahtab, Yasmin Adam, Amy Wise, Takwanisa Machemedza, Cynthia G. Whitney, the CHAMPS Network, A. S. M. Nawshad Uddin Ahmed, Mahbubul Hoque, Mohammed Kamal, Mohammad Mosiur, Ferdousi Begum, Saria Tasnim, Meerjady Sabrina Flora, Farida Arjuman, Iqbal Ansary Khan, Tahmina Shirin, Mahbubur Rahman, Sanwarul Bari, Shahana Parveen, Farzana Islam, Mohammad Zahid Hossain, Kazi Munisul Islam, Mohammad Sabbir Ahmed, K. Zaman, Mustafizur Rahman, Dilruba Ahmed, Md. Atique Iqbal Chowdhury, Muntasir Alam, Kyu Han Lee, Ferdousi Islam, Joseph O. Oundo, Fikremelekot Temesgen, Melisachew Mulatu Yeshi, Alexander M. Ibrahim, Tadesse Gure, Yunus Edris, Addisu Alemu, Dadi Marami, Ephrem Lemma, Ayantu Mekonnen, Henok Wale, Tseyon Tesfaye, Haleluya Leulseged, Tadesse Dufera, Anteneh Belachew, Fentabil Getnet, Surafel Fentaw, Yenework Acham, Stian M. S. Orlien, Mahlet Abayneh Gizaw, Emily Rogena, Florence Murila, Gunturu Revathi, Paul K. Mitei, Magdalene Kuria, Jennifer R. Verani, Aggrey Igunza, Peter Nyamthimba, Elizabeth oele, Karen D. Fairchild, Carol L. Greene, Rima Koka, Sharon M. Tennant, Ashka Mehta, J. Kristie Johnson, Adama Mamby Keita, Nana Kourouma, Uma U. Onwuchekwa, Awa Traore, Doh Sanogo, Diakaridia Sidibe, Seydou Sissoko, Diakaridia Kone, Milton Kindcardett, Khátia Munguambe, Ariel Nhacolo, Tacilta Nhampossa, Elisio Xerinda, Justina Bramugy, Celso Monjane, Sheila Nhachungue, Juan Carlos Hurtado, Maria Maixenchs, Clara Menéndez, Jaume Ordi, Natalia Rakislova, Marta Valente, Dercio Chitungo, Zara Manhique, Sibone Mocumbi, Fabiola Fernandes, Carla Carrilho, Rebecca Pass Philipsborn, Jeffrey P. Koplan, Mischka Garel, Betsy Dewey, Shailesh Nair, Navit T. Salzberg, Lucy Liu, Rebecca Alkis‐Ramirez, Jana M. Ritter, Sherif R. Zaki, Joy Gary, Jonas M. Winchell, Jacob Witherbee, Jessica L. Waller, Ruby Fayorsey, Ronita Luke, Ima‐Abasi Bassey, Dickens Kowuor, Foday Sesay, Baindu Kosia, Samuel Pratt, Carrie‐Jo Cain, Solomon Samura, Portia Mutevedzi, Fatima Solomon, Ashleigh Fritz, Noluthando Dludlu, Constance Ntuli, Richard Chawana, Karen Petersen, Sanjay G. Lala, Sithembiso Velaphi, Yasmin Adam, Jeannette Wadula, Martin Hale, Peter J. Swart, Hennie Lombaard, Gillian Sorour

**Affiliations:** ^1^ Rollins School of Public Health Emory University Atlanta Georgia USA; ^2^ Emory Global Health Institute Emory University Atlanta Georgia USA; ^3^ U.S. Military HIV Research Program Walter Reed Army Institute of Research Bethesda Maryland USA; ^4^ Center for Global Health US Centers for Disease Control and Prevention Atlanta Georgia USA; ^5^ International Centre for Diarrhoeal Disease Research Dhaka Bangladesh; ^6^ Bloomberg School of Public Health Johns Hopkins University Baltimore Maryland USA; ^7^ London School of Hygiene & Tropical Medicine London UK; ^8^ College of Health and Medical Sciences Haramaya University Harar Ethiopia; ^9^ Kisumu County Department of Health Kisumu Kenya; ^10^ US Centers for Disease Control and Prevention‐Kenya Kisumu Kenya; ^11^ US Centers for Disease Control and Prevention‐Kenya Nairobi Kenya; ^12^ KEMRI‐Wellcome Trust Research Programme Kilifi Kenya; ^13^ Centre pour le Développement des Vaccins Ministère de la Santé Bamako Mali; ^14^ School of Medicine University of Maryland Baltimore Maryland USA; ^15^ ISGlobal, Hospital Clínic Universitat de Barcelona Barcelona Spain; ^16^ Centro de Investigação em Saúde de Manhiça Maputo Mozambique; ^17^ Instituto Nacional de Saúde Maputo Mozambique; ^18^ Crown Agents Freetown Sierra Leone; ^19^ South African Medical Research Council Vaccines and Infectious Diseases Analytics Research Unit University of the Witwatersrand Johannesburg South Africa

**Keywords:** cause of death, child mortality, selection bias, South Asia, stillbirths, Sub‐Saharan Africa

## Abstract

**Background:**

Studies of child mortality that employ minimally invasive tissue sampling (MITS) produce highly accurate cause of death data; however, selection bias may render these as non‐representative of their underlying populations.

**Objectives:**

Estimate cause‐specific mortality fractions and rates for the five most frequent causes—underlying and others in the chain of events leading to death—among stillbirths, neonatal, infant and child deaths—in Sub‐Saharan Africa and South Asia, adjusted for any identified selection biases.

**Methods:**

The Child Health and Mortality Prevention Surveillance (CHAMPS) Network collects standardised, population‐based, longitudinal data on causes of death among stillbirths and under‐five children in 12 catchments in seven countries in Sub‐Saharan Africa and South Asia. Cause‐specific mortality fractions and rates were calculated for the five most frequent causes among stillbirths, neonatal, infant and child deaths, and for the five most frequent maternal conditions among perinatal deaths; all estimates were subsequently adjusted for selection bias. Selection probabilities were estimated from membership in subgroups defined by factors hypothesised to affect selection.

**Results:**

In 2017–2020, of 10,122 deaths ascertained, 5847 (57.8%) were enrolled in CHAMPS and 2654 (26.2%) additionally consented to MITS. Estimates were calculated for 265 and 65 site/age‐specific causes of death and maternal conditions, respectively; five (1.9%) and four (6.2%) required adjustment, respectively, but they did not meaningfully change. Estimates were calculated for 34 site‐specific causes of death among all stillbirths and under‐five deaths combined; 28 (82.4%) required adjustment (all included age at death), and change‐in‐estimates demonstrated considerable variability.

**Conclusions:**

Selection bias is not a concern in the CHAMPS Network. Deaths where MITS were performed accurately represent the distribution of causes of death in their respective target populations, specifically when stratified by age or adjusted accordingly. Future studies of child mortality that employ MITS should consider adjusting for age at death for their measures of frequency.

## 1 Introduction

Sub‐Saharan Africa and South Asia accounted for 77% of 2.0 million stillbirths [1] and 82% of 5.2 million under‐five deaths [2] worldwide in 2019. Current preventive efforts are informed by child mortality estimates based on vital registration systems, population censuses, verbal autopsies and household surveys—all of which are often incomplete and prone to systematic error [2, 3, 4]. Global estimates of child mortality aetiologies have traditionally relied on the underlying cause, neglecting other causes in the chain of events leading to death and omitting maternal conditions that may have precipitated or indirectly contributed to perinatal deaths [3, 5, 6, 7]. In response, the Child Health and Mortality Prevention Surveillance (CHAMPS) Network was established in 2015 to collect detailed, standardised, population‐based, longitudinal data within a network of sites in areas with high child mortality, with the overarching objectives of understanding and tracking preventable causes of stillbirths and under‐five deaths globally [3, 8].

CHAMPS employs minimally invasive tissue sampling (MITS) to facilitate post‐mortem pathology, microbiology, molecular and other diagnostic testing, including the identification of specific pathogens; however, not all those enrolled consent to this procedure [5, 9]. MITS has been shown to be less physically disruptive, faster, more culturally acceptable, less resource‐intensive and nearly as valid as a complete diagnostic autopsy for determining causes of death [3, 10, 11, 12, 13]. However, only a subset of all known eligible deaths in these areas enrol in CHAMPS, and an even smaller subset undergo MITS, as consent may not have been granted or burial may have occurred before the family was approached for enrolment [14]. Reasons for MITS refusal may include having had a negative experience with hospital care, concerns with disfiguring the body or delaying the funeral, feeling there is no need for further examination and religious or cultural concerns [15, 16, 17, 18]. Families of deceased children who do not consent to MITS tend to be less educated and less likely to be employed [16]. Moreover, infant and child deaths and deaths that occur in the community tend to be underrepresented in CHAMPS compared to stillbirths and neonatal deaths and in‐hospital deaths, possibly due to issues of logistics and feasibility [14, 19]. Cause‐specific mortality estimates that rely solely on deaths where MITS were performed may therefore not accurately represent their underlying target populations [20]. Selection bias is introduced when deaths where MITS were and were not performed differ in terms of characteristics that affect both selection and the outcome of interest; these associations must be independently assessed for each exposure‐outcome pair [21, 22]. Previously published data from CHAMPS have reported cause‐specific mortality frequencies or fractions that only represent deaths where MITS were performed, without adjusting for possible selection bias and extrapolating to all deaths in the catchment areas [3, 5].

Objectives of this current work include: (1) estimating crude mortality fractions and rates for the five most frequent causes of death—underlying and others in the chain of events leading to death—among stillbirths, neonatal, infant and child deaths in each site; (2) estimating crude fractions and rates for the five most frequent maternal conditions for stillbirths and neonatal deaths in each site; (3) adjusting all estimates for any identified selection biases so that they are representative of their target populations; and (4) comparing adjusted mortality fractions and rates with those published in the scientific literature.

## 2 Methods

### 2.1 Mortality and Demographic Surveillance

CHAMPS is an ongoing study conducted in 12 catchments in seven countries in Sub‐Saharan Africa and South Asia: Baliakandi and Faridpur, Bangladesh; Haramaya, Harar and Kersa, Ethiopia; Manyatta and Siaya, Kenya; Bamako, Mali; Manhiça and Quelimane, Mozambique; Makeni, Sierra Leone; and Soweto, South Africa. Site characteristics and selection criteria have been described elsewhere [14]. Of note, not all CHAMPS sites began enrolment at the same time; Mozambique began in 2016; South Africa, Kenya, Mali and Bangladesh began in 2017; and Sierra Leone and Ethiopia began in 2019. Most catchments—except Faridpur, Bangladesh; Quelimane, Mozambique; and Makeni, Sierra Leone—carry out mortality surveillance within a demographic surveillance system (DSS) that captures sociodemographic characteristics and data on births, deaths, pregnancies and in‐ and out‐migration episodes within a geographically defined area [23, 24, 25, 26]. Eligible deaths captured in the DSS but never enrolled in CHAMPS are referred to as ‘DSS only deaths’. Ethics committees overseeing investigators at each site and at Emory University (Atlanta, GA, USA) approved overall and site‐specific protocols, as appropriate.

According to CHAMPS procedures, attempts were made to notify staff of stillbirths and under‐five deaths within the first 24 h. Shortly thereafter, CHAMPS staff approached families for eligibility screening. All stillborn foetuses and deceased children who resided within the catchment were eligible for inclusion. Families provided consent for MITS [27], verbal autopsy (VA) [28] and clinical data abstraction. MITS eligibility required that CHAMPS staff were notified within 24 h of death (or ≤ 72 h if post‐mortem refrigeration was used) and that the body of the stillborn foetus or deceased child was available for specimen collection. Families of stillborn foetuses and deceased children who were not eligible for the MITS procedure were asked to consent for only the VA interview and clinical data abstraction. Deaths enrolled in CHAMPS whose families have and have not granted consent for MITS are referred to as MITS and non‐MITS cases, respectively.

### 2.2 Specimen and Data Collection

All deaths between 1 January 2017 and 31 December 2020 were included in the current analysis. Methods used for specimen and data collection for the MITS procedure have been described previously [3]. Briefly, trained staff took photographs and anthropometric measurements before specimen collection. Tissue specimens were collected from the lungs, heart, brain, liver and bone marrow; peripheral blood, cerebrospinal fluid, stool and nasopharyngeal secretions were also collected [27]. Blood samples were tested for HIV DNA or RNA by PCR and for malaria using thick and thin smears and rapid diagnostic tests; blood and cerebrospinal fluid underwent culture for bacteria. Five custom TaqMan Array Cards (TAC; ThermoFisher Scientific, Waltham, MA, USA) with specific molecular assays were used to detect 116 pathogen targets [27, 29]. Tissues were examined using histopathology techniques, including routine and special stains [30]. Data were abstracted from all available clinical records of deceased children, and relevant maternal health records were abstracted for stillbirths and neonatal deaths. Families were interviewed using translations of the 2016 World Health Organization (WHO) VA instrument [14, 28].

### 2.3 Cause of Death Determination

A complete description of the determination of cause of death (DeCoDe) process has been described elsewhere [3, 6]. Briefly, all available data for each case were reviewed by DeCoDe panels convened at each site, consisting of paediatricians, obstetricians, epidemiologists, pathologists, microbiologists and other subject‐matter experts [6]. The panels reviewed available case data and determined the chain of events (immediate, underlying, comorbid causes—hereafter referred to as the causal chain) leading to death and assigned WHO International Classification of Diseases, Tenth Revision (ICD‐10) codes [31]. Panels also identified and assigned maternal conditions contributing to perinatal deaths using WHO ICD‐Perinatal Mortality (PM) codes [32]. Causes and maternal conditions were grouped according to the Global Burden of Disease (GBD) categories for analysis [33]. MITS cases that have been reviewed by a DeCoDe panel and assigned a cause of death are referred to as DeCoDed MITS cases.

### 2.4 Statistical Analysis

Details of the analytic method have been described elsewhere [34]. Descriptive analyses were performed to characterise all ascertained stillbirths and under‐five deaths in the DSS areas by whether the deaths were enrolled in CHAMPS and whether MITS were performed. Factors of interest included: site of death; age at death (stillbirth [no spontaneous breathing or movement at time of delivery and (1) weighing > 1 kg and/or (2) estimates gestational age ≥ 28 weeks], neonate [0–28 days], infant [29–364 days] or child [1–5 years]); sex at birth (male or female); location of death (community or facility); season of death (dry or rainy); VA cause of death (Inter‐VA [35]: infection, trauma or other); and maternal education (none, primary, secondary or tertiary). If DSS data were available, it is assumed that all CHAMPS cases were also captured in the DSS.

The five most frequent underlying causes and causes anywhere in the causal chain were identified by age group (including stillbirths and under‐five deaths combined—hereafter referred to as total under‐five [36]) and site, and the five most frequent maternal conditions were identified for stillbirths and neonates by site. Cause‐specific mortality fractions (CSMF) were calculated for each of the five most frequent causes or maternal conditions, by age group and site. Crude CSMFs (cCSMF) were calculated as the proportion of age‐specific deaths attributed to each cause among all age‐specific DeCoDed MITS cases. Selection bias was hypothesised to be the greatest threat to the external validity of CHAMPS MITS cases to the underlying target populations; other biases were not explored. To calculate adjusted CSMFs (aCSMF), factors hypothesised to affect selection (age at death, sex at birth, location of death, season of death, VA cause of death and maternal education) had to meet four a priori criteria for adjustment: (1) associated with MITS consent; (2) missing < 20% data when comparing MITS and non‐MITS cases; (3) associated with the specific cause of death; and (4) missing < 20% data when comparing cause‐specific deaths and deaths due to all other causes. Factors were selected for adjustment if one or at most two factors (where age at death must be one of the two, due to data limitations) met all four criteria. If three or more factors met all four criteria, the top two were selected based on the following a priori hierarchy: age at death, season of death, location of death, VA cause of death, sex at birth and maternal education. Age‐specific CHAMPS cases (non‐MITS and MITS), MITS cases and all deaths in the target population were stratified by the factors that met selection. Selection probabilities were calculated as the proportion of age‐specific MITS cases among all eligible age‐specific deaths in the target population for each stratum. Direct standardisation was then performed for factors that had met all four criteria. The target population for most sites was all eligible age‐specific deaths ascertained in the DSS in each respective site. However, for sites where DSS data were unavailable for one or more catchments, the target population consisted of all age‐specific CHAMPS deaths regardless of MITS consent.

Crude and adjusted cause‐specific mortality rates (cCSMR and aCSMR, respectively) were calculated as the product of the cCSMF or aCSMF and the all‐cause age‐specific mortality rate, respectively, for each of the five most frequent causes or maternal conditions. Where DSS data were available, the all‐cause age‐specific mortality rate in the target population was calculated as the number of age‐specific deaths among all live‐births (and stillbirths, when appropriate) [37]. Where DSS data were unavailable (Mali and Sierra Leone), the all‐cause age‐specific mortality rate in the target population was sourced from the Demographic and Health Surveys (DHS) Program [38], in which case the all‐cause age‐specific mortality rate may represent a larger geographic region than the catchment itself, and the year of data collection may not exactly coincide with that of CHAMPS.

We calculated 90% Bayesian credible intervals (CrI) based on a non‐informative prior distribution for all estimates. Analyses were performed in R 4.2.1 (R Core Team, Vienna, Austria) [39]. An R package called champsmortality was specifically developed and validated to help calculate these estimates [40]. All research conformed to the principles embodied in the Declaration of Helsinki.

### 2.5 Missing Data

Among all ascertained deaths in the DSS and CHAMPS, 1.0% were missing site of death (DSS only deaths, non‐MITS cases and MITS cases: 2.4%, 0.0% and 0.0%, respectively), 7.2% were missing sex at birth (16.1%, 0.8% and 0.2%, respectively), 11.4% were missing location of death (26.0%, 0.7% and 0.0%, respectively), 1.8% were missing season of death (4.1%, 0.0% and 0.0%, respectively), 43.3% were missing VA cause of death (78.5%, 16.3% and 17.8%, respectively), and 53.6% were missing maternal education (34.7%, 61.2% and 75.4%, respectively).

### 2.6 Ethics Approval

Ethics committees overseeing investigators at each site and at Emory University (Atlanta, GA, USA) approved overall and site‐specific protocols, as appropriate. The U.S. Centers for Disease Control and Prevention (CDC; Atlanta, GA, USA) relied on ethical review committees at Emory University and at individual sites where CDC staff were directly engaged to review the protocol. All research conformed to the principles embodied in the Declaration of Helsinki.

## 3 Results

### 3.1 Enrolment and Consent

A total of 15,106 unique death notifications were received in 2017–2020, of which 7844 (51.9%) were deemed eligible, 6968 (46.1%) were requested to consent and 5847 (38.7%) enrolled in CHAMPS. Of those enrolled, 3024 (51.7%) were requested to consent for MITS, 2707 (46.3%) granted consent, 2654 (45.4%) had the procedure performed and 2547 (43.6%) have been DeCoDed. The analytic sample consists of 10,122 deaths: 4275 DSS only deaths and 5847 deaths enrolled in CHAMPS (Figure S1). Venn diagrams of the proportions of eligible deaths in the DSS enrolled in CHAMPS and consented to MITS are depicted by site and age group in Figure S2.

### 3.2 Sample Characteristics

Most CHAMPS cases were stillbirths (33.6%) or neonates (36.7%), male (55.2%), died in a facility (74.8%), died during the dry season (57.3%), had a VA cause of death other than infection or trauma (77.6%) and were born to mothers educated at the primary level (40.5%; Table 1). DSS only deaths were predominantly children (39.5%), male (55.3%), died in the community (54.4%), during the dry season (54.0%), had a VA cause of death other than infection or trauma (52.9%) and were born to mothers educated at the secondary level (36.8%).

**TABLE 1:** Characteristics of all ascertained stillbirths and under‐five deaths in demographic surveillance systems by CHAMPS enrolment and MITS performed (2017–2020).

	DSS only^a^	CHAMPS	
		Non‐MITS^b^	MITS^c^
	*N* = 4380	*N* = 3193	*N* = 2654
	*n* (%)^d^	*n* (%)^d^	*n* (%)^d^
Case characteristics			
Site of death^e^			
Bangladesh	161 (3.8)	918 (28.8)	212 (8.0)
Ethiopia	1156 (27.0)	223 (7.0)	173 (6.5)
Kenya	1394 (32.6)	185 (5.8)	500 (18.8)
Mali^f^	—	839 (26.3)	194 (7.3)
Mozambique	1289 (30.2)	581 (18.2)	646 (24.3)
Sierra Leone^g^	—	303 (9.5)	229 (8.6)
South Africa	275 (6.4)	144 (4.5)	700 (26.4)
Age at death^e^, ^f^, ^g^			
Stillbirths	718 (16.4)	1124 (35.2)	838 (31.6)
Neonates	875 (20.0)	1058 (33.1)	1087 (41.0)
Infants	1059 (24.2)	535 (16.8)	382 (14.4)
Children	1728 (39.5)	476 (14.9)	347 (13.1)
Sex at birth^e^			
Male	2034 (55.3)	1737 (54.8)	1476 (55.7)
Female	1642 (44.7)	1431 (45.2)	1174 (44.3)
Location of death^e^			
Community	1763 (54.4)	1170 (36.9)	299 (11.3)
Facility	1477 (45.6)	2002 (63.1)	2354 (88.7)
Season of death^e^, ^h^			
Dry	2267 (54.0)	1747 (54.7)	1605 (60.5)
Rainy	1932 (46.0)	1446 (45.3)	1049 (39.5)
Verbal autopsy cause of death^e^, ^i^			
Infection	410 (43.6)	559 (20.9)	430 (19.7)
Trauma	33 (3.5)	75 (2.8)	25 (1.1)
Other	497 (52.9)	2040 (76.3)	1728 (79.2)
Maternal characteristics^j^			
Education^g^			
None	809 (28.3)	307 (24.8)	110 (16.9)
Primary	918 (32.1)	509 (41.1)	256 (39.3)
Secondary	1053 (36.8)	339 (27.4)	184 (28.2)
Tertiary	79 (2.8)	83 (6.7)	102 (15.6)

Abbreviations: CHAMPS, Child Health and Mortality Prevention Surveillance Network; DSS, demographic surveillance system; ICD‐10, International Classification of Diseases, Revision 10; MITS, minimally invasive tissue sampling; VA, verbal autopsy.

^a^ Eligible stillbirths and under‐five deaths captured in the DSS but never enrolled in CHAMPS.

^b^ Stillbirths and under‐five deaths enrolled in CHAMPS but for whom MITS was not performed.

^c^ Stillbirths and under‐five deaths enrolled in CHAMPS and for whom MITS was performed.

^d^ Percentages (column distributions) may not sum to 100% due to rounding.

^e^ Missing: [DSS only]: site (*n* = 105), age at death (*n* = 0), sex at birth (*n* = 704), location of death (*n* = 1140), season of death (*n* = 181), VA cause of death (*n* = 3440), maternal education (*n* = 1521); [Non‐MITS]: sex at birth (*n* = 25), location of death (*n* = 21), VA cause of death (*n* = 519), maternal education (*n* = 1955); [MITS]: sex at birth (*n* = 4), location of death (*n* = 1), VA cause of death (*n* = 471), maternal education (*n* = 2002).

^f^ [Mali]: DSS data included in non‐MITS; [Sierra Leone]: no DSS data available.

^g^ Stillbirths (no spontaneous breathing or movement at time of delivery and [1] weighing > 1 kg and/or [2] estimated gestational age ≥ 28 weeks); neonates (0–28 days); infants (29–365 days); children (1–5 years).

^h^ Dry, rainy: BD (November–May, June–October); ET (October–May, June–September); KE (July and December–March, April–June and August–November); ML (November–May, June–October); MZ (May–October, November–April); SL (October–May, June–September); ZA (March–November, December–February).

^i^ Inter‐VA algorithm: Infection (ICD‐10 codes 01, 10.3–10.5); trauma (ICD‐10 code 12).

^j^ All characteristics pertinent to time of pregnancy.

### 3.3 Stillbirths

**FIGURE 1:**
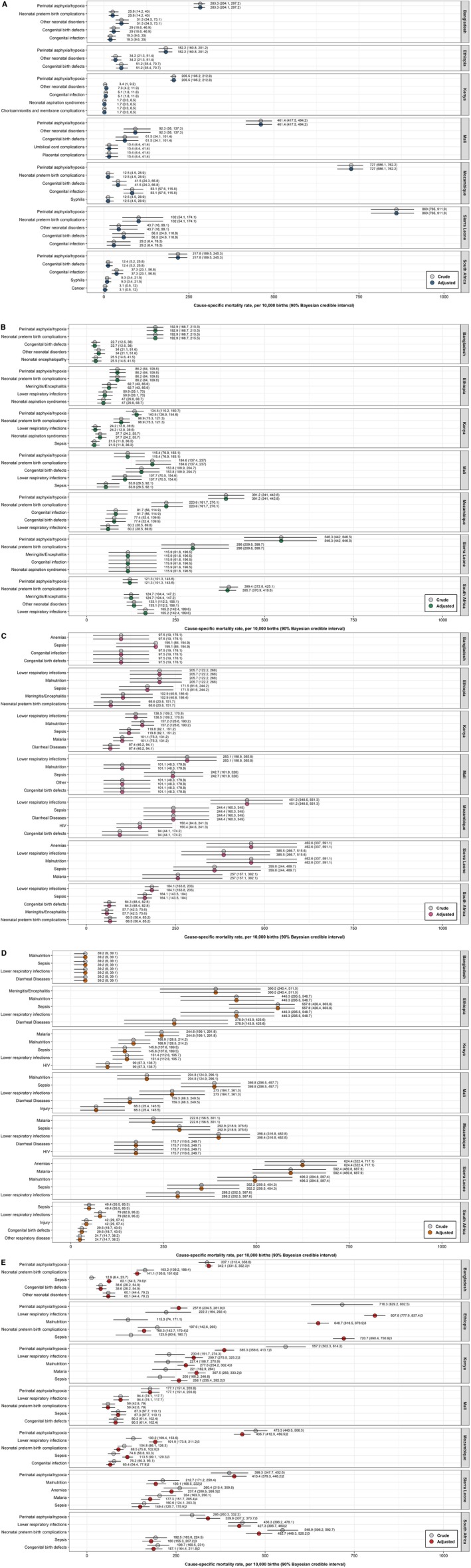
Rates of the most frequent perinatal and paediatric causes of death in the CHAMPS Network, 2017–2020. Estimates are adjusted for selection bias due to enrolment and post‐mortem sampling. (A) Stillbirths, no spontaneous breathing or movement at time of delivery and (1) weighing > 1 kg and/or (2) estimated gestational age ≥ 28 weeks. (B) Neonates (0–28 days). (C) Infants (29–365 days). (D) Children (1–5 years). (E) Under‐five, includes stillbirths, neonates, infants and children. CHAMPS, Child Health and Mortality Prevention Surveillance Network.

**FIGURE 2:**
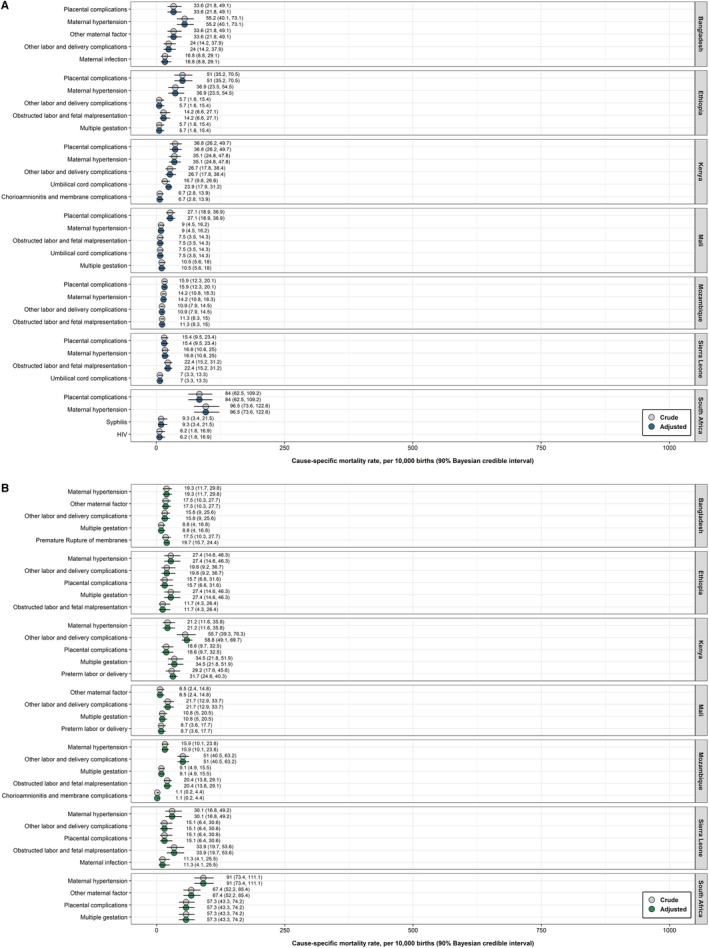
Rates of the most frequent contributing maternal conditions in the CHAMPS Network, 2017–2020. Estimates are adjusted for selection bias due to enrolment and post‐mortem sampling. (A) Stillbirths, no spontaneous breathing or movement at time of delivery and (1) weighing > 1 kg and/or (2) estimated gestational age ≥ 28 weeks. (B) Neonates (0–28 days). CHAMPS, Child Health and Mortality Prevention Surveillance Network.

Regarding cause‐specific mortality, the criteria for adjustment were never met for any site (Figure 1A and Figure S3A). Regarding contributing maternal conditions, the criteria for adjustment for MITS ascertainment were only met for umbilical cord complications in Kenya (crude fraction [90% CrI] = 7.5% [4.4, 11.9], adjusted fraction [90% CrI] = 10.7% [8.0, 13.9], change‐in‐estimate (CIE) = 3.2%; crude rate [90% CrI] = 1.8 per 1000 births [1.1, 2.9], adjusted rate [90% CrI] = 2.6 per 1000 births [1.9, 3.3], CIE = 0.8; adjusted for location of death; Figure 2A and Figure S4A).

### 3.4 Neonates

Regarding cause‐specific mortality, the criteria for adjustment were only met for perinatal asphyxia/hypoxia in Kenya (cCSMF [90% CrI] = 34.0% [27.9, 40.6], aCSMF [90% CrI] = 35.5% [32.1, 39.1], CIE = 1.5%; cCSMR [90% CrI] = 6.5 per 1000 live‐births [5.4, 7.8], aCSMR [90% CrI] = 6.8 per 1000 live‐births [6.2, 7.5], CIE = 0.3; adjusted for season of death) and neonatal preterm birth complications in South Africa (cCSMF [90% CrI] = 62.2% [58.1, 66.2], aCSMF [90% CrI] = 61.6% [57.8, 65.4], CIE = −0.6%; cCSMR [90% CrI] = 26.6 per 1000 live‐births [24.8, 28.3], aCSMR [90% CrI] = 26.3 per 1000 live‐births [24.7, 27.9], CIE = −0.3; adjusted for location of death; Figure 1B and Figure S3B). Regarding contributing maternal conditions, the criteria for adjustment were only met for premature rupture of membranes in Bangladesh (crude fraction [90% CrI] = 9.2% [5.4, 14.5], CIE = 1.1%; crude rate [90% CrI] = 2.2 per 1000 live‐births [1.3, 3.5], CIE = 0.3; adjusted for season of death), other labour and delivery complications in Kenya (crude fraction [90% CrI] = 14.1% [9.9, 19.3], adjusted fraction [90% CrI] = 14.9% [12.4, 17.6], CIE = 0.8%; crude rate [90% CrI] = 3.4 per 1000 live‐births [2.4, 4.6], adjusted rate [90% CrI] = 3.6 per 1000 live‐births [3.0, 4.2], CIE = 0.2; adjusted for season of death) and preterm labour or delivery in Kenya (crude fraction [90% CrI] = 7.4% [4.5, 11.5], CIE = 0.6%; crude rate [90% CrI] = 1.8 per 1000 live‐births [1.1, 2.8], CIE = 0.1; adjusted for season of death; Figure 2B and Figure S4B).

### 3.5 Infants and Children

Criteria for adjustment were never met for infants (Figure 1D and Figure S3D) or children (Figure 1D and Figure S3D) for any site.

### 3.6 Total Under‐Five

Twenty‐eight out of 34 (82.4%) causes examined met the criteria for adjustment; of which, 15 (53.6) were adjusted for age at death and location of death, 10 (35.7%) were adjusted for age at death only, 2 (7.1%) were adjusted for age at death and VA cause of death, and 1 (3.6%) was adjusted for age at death and season of death (Figure 1E and Figure S3E). The largest CSMF and CSMR CIE occurred for sepsis in Ethiopia (cCSMF [90% CrI] = 9.0% [5.9, 13.2], aCSMF [90% CrI] = 52.7% [50.5, 54.9], CIE = 43.7%; cCSMR [90% CrI] = 3.8 per 1000 births [2.5, 5.6], aCSMR [90% CrI] = 22.5 per 1000 births [21.6, 23.4], CIE = 18.7; adjusted for age at death and location of death). On average, adjustment increased the CSMF by 3.4% (standard deviation [SD] = 15.6, range = [−33.6, 43.7]) and increased the CSMR by 1.4 per 1000 births (SD = 6.6, range = [−14.4, 18.7]).

## 4 Comment

### 4.1 Principal Findings

The work presented represents a robust analysis to extrapolate age‐specific fractions and rates of cause‐specific mortality, and of maternal conditions, from non‐representative samples of DeCoDed MITS cases to larger target populations. Previous estimates from CHAMPS and other studies that employ MITS have reported cause‐specific mortality frequencies or fractions that only represent MITS cases, without adjusting for selection bias [3, 5, 41, 42, 43, 44]. That said, the results here suggest that such an adjustment may not always be necessary, that crude estimates are often just as valid, particularly when stratified by age [20, 45].

### 4.2 Strengths of the Study

Strengths of this study include its standardised, population‐based, longitudinal approach; employment of MITS for post‐mortem evaluation; standardised DeCoDe process; determination of not only the underlying cause, but also the causal chain; determination of the main maternal conditions; use of DSS data to characterise deaths never enrolled in CHAMPS; and application of a method to extrapolate sparse gold standard cause of death data to characterise broader catchment areas, thereby addressing the issue of external validity, a common criticism of CHAMPS data.

### 4.3 Limitations of the Data

Limitations of this study include unavailable or discrepant DSS data for some sites; all‐cause mortality rates sourced from DHS when DSS data were unavailable, which may not coincide geographically or temporally with CHAMPS; sparse data, particularly for uncommon outcomes; underlying assumptions—missing at random, ad‐hoc criteria to identify factors for adjustment, no residual bias due to categorisation of age, hierarchy of factors when more than 2 factors met criteria, no unmeasured factors that affect selection and all CHAMPS deaths are a subset of DSS deaths—presumed true; and extrapolation beyond the target population was not performed, therefore adjusted estimates do not represent the entire site.

### 4.4 Interpretation

For the major age‐specific underlying and causal chain causes of death in each site, there were only five instances where adjustment for selection bias was necessary; even still, both the CSMF and CSMR did not meaningfully change. For the major age‐specific maternal conditions in each site, there were only four instances where adjustment for selection bias was necessary; even still, both the CSMF and CSMR did not meaningfully change. In contrast, for the major stillbirth and under‐five causal chain causes of death in each site, there were 53 instances where adjustment for selection bias was necessary, all of which included age at death; here, we find considerable variability in the change of both CSMF and CSMR.

The major country‐level causes of death among neonatal, infant and child deaths reported by the Institute for Health Metrics and Evaluation (IHME) GBD Study [2, 46, 47] are mostly consistent with those reported here, in that both report them as highly frequent causes and, although their point estimates do not correspond exactly, they have overlapping measures of uncertainty. Notably, results are less consistent for infant and child deaths. This discrepancy, however, is likely because some causes are more likely to be attributed as an immediate or comorbid cause of death among infants and children, not necessarily an underlying cause, and therefore would not be captured in the IHME GBD data. Estimates for CSMRs published by the WHO Global Health Observatory [48, 49, 50] (GHO) are also largely consistent with those reported here, although comparisons are difficult as they do not provide a measure of uncertainty. And yet, considerable differences also exist. Again, this inconsistency is likely because the WHO GHO data do not capture immediate or comorbid causes of death. Thus, due to differing methodological approaches, operational definitions, and geographical representations, direct comparisons should be made with abundant caution, if at all.

## 5 Conclusions

The CHAMPS Network can better inform and help target preventive efforts to curtail child mortality in Sub‐Saharan Africa and South Asia. The work presented here has shown that MITS cases appear to accurately represent the distribution of causes of death in their respective underlying target populations, when stratified by age or otherwise adjusted accordingly. Future studies of child mortality that employ MITS should consider adjusting for age at death for their measures of frequency. Moreover, efforts should be made to further extrapolate these CHAMPS data so inferences can be made at the national level.

## Author Contributions

D.M.B., S.E.A., E.S.G., J.A.G.S., N.A., D.O., V.A., S.O.S., K.L.K., Q.B., I.M., I.O. and S.Madhi designed the protocol and led the involvement of sites in the CHAMPS Network. A.I.C., K.M.I., A.R., L.M., Y.A.A., Y.Y.A., D.O., B.A.T.‐B., G.A., M.D.T., A.M.K., K.C., A.N., C.S., D.K., B.D., A.M., J.S.S., S.M., Y.A.A., A.W. and T.M. helped coordinate and implement CHAMPS procedures in the sites. K.J.V., J.A.M., Z.J.M., P.M.G. and C.G.W. conceptualised the manuscript. K.J.V., J.A.M., Z.J.M. and P.M.G. directed data management, conducted data analyses and directly accessed and verified the underlying data reported in the manuscript. K.J.V. drafted the manuscript. All authors revised the manuscript. K.J.V., J.A.M., Z.J.W., P.M.G., D.M.B. and C.G.W. had final responsibility for the decision to submit it for publication. K.J.V. is the corresponding author and guarantor for this manuscript. All authors have reviewed this manuscript and have approved the decision to submit it for publication.

## Conflicts of Interest

The authors declare no conflicts of interest.

## Supporting information


**Figure S1:** Flow diagram from ascertainment to CHAMPS enrolment, MITS performed and cause of death determination, by site. DSS only refers to deaths captured in the DSS but never enrolled in CHAMPS; non‐MITS refers to deaths enrolled in CHAMPS but for whom MITS was not performed; MITS refers to deaths enrolled in CHAMPS and for whom MITS was performed; DeCoDed refers to deaths for whom MITS was performed and reviewed by the DeCoDe panel as of 24 May 2022. All‐cause age‐specific mortality rates from the DHS were substituted during calculations for catchments without DSS data availability. CHAMPS, Child Health and Mortality Prevention Surveillance Network; children (1–5 years), DeCoDe, determination of cause of death; DHS, Demographic and Health Surveys Program; DSS, demographic surveillance system; infants (29–365 days); MITS, minimally invasive tissue sampling; neonates, neonates (0–28 days); stillbirths (no spontaneous breathing or movement at time of delivery and [1] weighing > 1 kg and/or [2] estimated gestational age ≥ 28 weeks).
**Figure S2:** Venn diagrams of enrolment and MITS performed among all ascertained deaths in the CHAMPS Network, by site and age. In sites with available DSS data, it is assumed all CHAMPS cases are also captured in the DSS system. (1) DSS data are ignored due to discordant availability among catchments. (2) DSS data included in count of non‐MITS CHAMPS cases. (3) DSS data are not available. (4) Stillbirths (no spontaneous breathing or movement at time of delivery and [1] weighing > 1 kg and/or [2] estimated gestational age ≥ 28 weeks); neonates (0–28 days); infants (29–365 days); children (1–5 years). (5) Combined for all catchments with available DSS data. CHAMPS, Child Health and Mortality Prevention Surveillance Network; DSS, demographic surveillance system; MITS, minimally invasive tissue sampling.
**Figure S3:** Fractions for the most frequent perinatal and paediatric causes of death in the CHAMPS Network, 2017–2020. (A) Stillbirths, no spontaneous breathing or movement at time of delivery and (1) weighing > 1 kg and/or (2) estimated gestational age ≥ 28 weeks. (B) Neonates (0–28 days). (C) Infants (29–365 days). (D) Children (1–5 years). (E) Under‐five, includes stillbirths, neonates, infants and children. CHAMPS, Child Health and Mortality Prevention Surveillance Network.
**Figure S4:** Fractions for the most frequent contributing maternal conditions in the CHAMPS Network, 2017–2020. (A) Stillbirths, no spontaneous breathing or movement at time of delivery and (1) weighing > 1 kg and/or (2) estimated gestational age ≥ 28 weeks. (B) Neonates (0–28 days). CHAMPS, Child Health and Mortality Prevention Surveillance Network.

## Data Availability

Summarised data are publicly available through the CHAMPS website: https://champshealth.org/data/enrolled‐population‐summary/. Requests for further detailed data, for research and evaluation purposes, can be made at: https://champshealth.org/data/.

## CHAMPS Network

*CHAMPS Bangladesh*—A. S. M. Nawshad Uddin Ahmed and Mahbubul Hoque of Dhaka Shishu Hospital and Institute; Mohammed Kamal, Mohammad Mosiur and Ferdousi Begum of Bangabandhu Sheikh Mujib Medical University; Saria Tasnim of Dhaka Community Medical College and Hospital; Meerjady Sabrina Flora of the Directorate General of Health Services in Bangladesh; Farida Arjuman of Institute of Cancer Research and Hospital, Iqbal Ansary Khan, Tahmina Shirin and Mahbubur Rahman of Institute of Epidemiology, Disease Control and Research; Sanwarul Bari, Shahana Parveen, Farzana Islam, Mohammad Zahid Hossain, Kazi Munisul Islam, Mohammad Sabbir Ahmed, K. Zaman, Mustafizur Rahman, Dilruba Ahmed, Md. Atique Iqbal Chowdhury, Muntasir Alam of International Centre for Diarrhoeal Disease Research, Bangladesh; Kyu Han Lee of the Department of Epidemiology, Johns Hopkins Bloomberg School of Public Health; and Ferdousi Islam of Popular Medical College and Hospital in Dhaka, Bangladesh. *CHAMPS Ethiopia*—Joseph O. Oundo of London School of Hygiene & Tropical Medicine; Fikremelekot Temesgen of Addis Ababa University in Addis Ababa, Ethiopia; Melisachew Mulatu Yeshi of Mekelle University, Mekele, Ethiopia; Alexander M. Ibrahim, Tadesse Gure, Yunus Edris, Addisu Alemu, Dadi Marami, Ephrem Lemma, Ayantu Mekonnen, Henok Wale, Tseyon Tesfaye, Haleluya Leulseged, Tadesse Dufera, Anteneh Belachew of the College of Health and Medical Sciences at Haramaya University; Fentabil Getnet, Surafel Fentaw, Yenework Acham of the Ethiopian Public Health Institute; Stian M. S. Orlien of the University of Hargeisa, Somaliland and Vestfold Hospital Trust, Tønsberg, Norway; and Mahlet Abayneh Gizaw of St. Paul's Hospital Millennium Medical College in Addis Ababa, Ethiopia. *CHAMPS Kenya*—Emily Rogena of Jomo Kenyatta University of Agriculture and Technology; Florence Murila of University of Nairobi; Gunturu Revathi of University of Aga Khan Medical College; Paul K. Mitei and Magdalene Kuria of Kisumu County Department of Health; and Jennifer R. Verani of the National Center for Immunization and Respiratory Diseases Branch at the Centers for Disease Control and Prevention; Aggrey Igunza of Kenya Medical Research Institute; Peter Nyamthimba of Kenya Medical Research Institute; Elizabeth oele of Kisumu County Health Department, Kisumu Kenya. *CHAMPS Mali*—Karen D. Fairchild of University of Virginia; Carol L. Greene, Rima Koka, Sharon M. Tennant, Ashka Mehta and J. Kristie Johnson of University of Maryland School of Medicine; Tatiana Keita of Clinique Pasteur in Bamako Mali; Adama Mamby Keita, Nana Kourouma, Uma U. Onwuchekwa, Awa Traore, Doh Sanogo, Diakaridia Sidibe and Seydou Sissoko of Centre pour le Développement des Vaccins; and Diakaridia Kone of CSRef Commune I in Bamako, Mali. *CHAMPS Mozambique*—Milton Kindcardett, Khátia Munguambe, Ariel Nhacolo, Tacilta Nhampossa, Elisio Xerinda and Justina Bramugy of Centro de Investigação em Saúde de Manhiça in Maputo; Celso Monjane and Sheila Nhachungue of Instituto Nacional de Saúde in Maputo; Juan Carlos Hurtado, Maria Maixenchs, Clara Menéndez, Jaume Ordi, Natalia Rakislova and Marta Valente of ISGlobal Hospital Clinic at Universitat de Barcelona; Dercio Chitungo and Zara Manhique of Quelimane Central Hospital; Sibone Mocumbi, Fabiola Fernandes and Carla Carrilho of Eduardo Mondlane University and Maputo Central Hospital. *CHAMPS Program Office*—Rebecca Pass Philipsborn of Emory University and Children's Healthcare of Atlanta; Jeffrey P. Koplan, Mischka Garel and Betsy Dewey of Emory Global Health Institute; Shailesh Nair, Navit T. Salzberg and Lucy Liu of the Public Health Informatics Institute at the Task Force for Global Health in Atlanta, Georgia, USA. *CDC Central Pathology Lab*—Rebecca Alkis‐Ramirez of Center for Global Health at the Centers for Disease Control and Prevention; Jana M. Ritter, Sherif R. Zaki and Joy Gary of Infectious Diseases Pathology Branch, National Center for Emerging and Zoonotic Diseases at the Centers for Disease Control and Prevention. *CDC TaqMan Array Team*—Jonas M. Winchell, Jacob Witherbee and Jessica L. Waller of the National Center for Immunization and Respiratory Diseases at the Centers for Disease Control and Prevention. *CHAMPS Sierra Leone*—Ruby Fayorsey of ICAP at Columbia University and Harlem Hospital Centers; Ronita Luke of Ministry of Health and Sanitation, Sierra Leone; Ima‐Abasi Bassey and Dickens Kowuor of Crown Agents; Foday Sesay of Sierra Leone's Ministry of Health & Sanitation; Baindu Kosia and Samuel Pratt of FOCUS 1000; Carrie‐Jo Cain and Solomon Samura of World Hope International. *CHAMPS South Africa*—Portia Mutevedzi, Fatima Solomon, Ashleigh Fritz, Noluthando Dludlu, Constance Ntuli and Richard Chawana of South African Council Vaccines and Infectious Diseases Analytics Research Unit, University of Witwatersrand; Karen Petersen, Sanjay G. Lala, Sithembiso Velaphi and Yasmin Adam of Chris Hani Baragwanath Academic Hospital and University of Witwatersrand; Jeannette Wadula, Martin Hale and Peter J. Swart of National Health for Laboratory Service in South Africa; Hennie Lombaard of Rahima Moosa Mother and Child Hospital; and Gillian Sorour of Wits Health Consortium.
