# Supplementary figures and images for: Major Causes of Perinatal and Paediatric Mortality in Sub‐Saharan Africa and South Asia: Adjustment for Selection Bias in the CHAMPS Network
Source: Paediatr Perinat Epidemiol. 2025 Sep 4;39(8):698–710. doi: 10.1111/ppe.70067 (PMC12658314; doi:10.1111/ppe.70067)

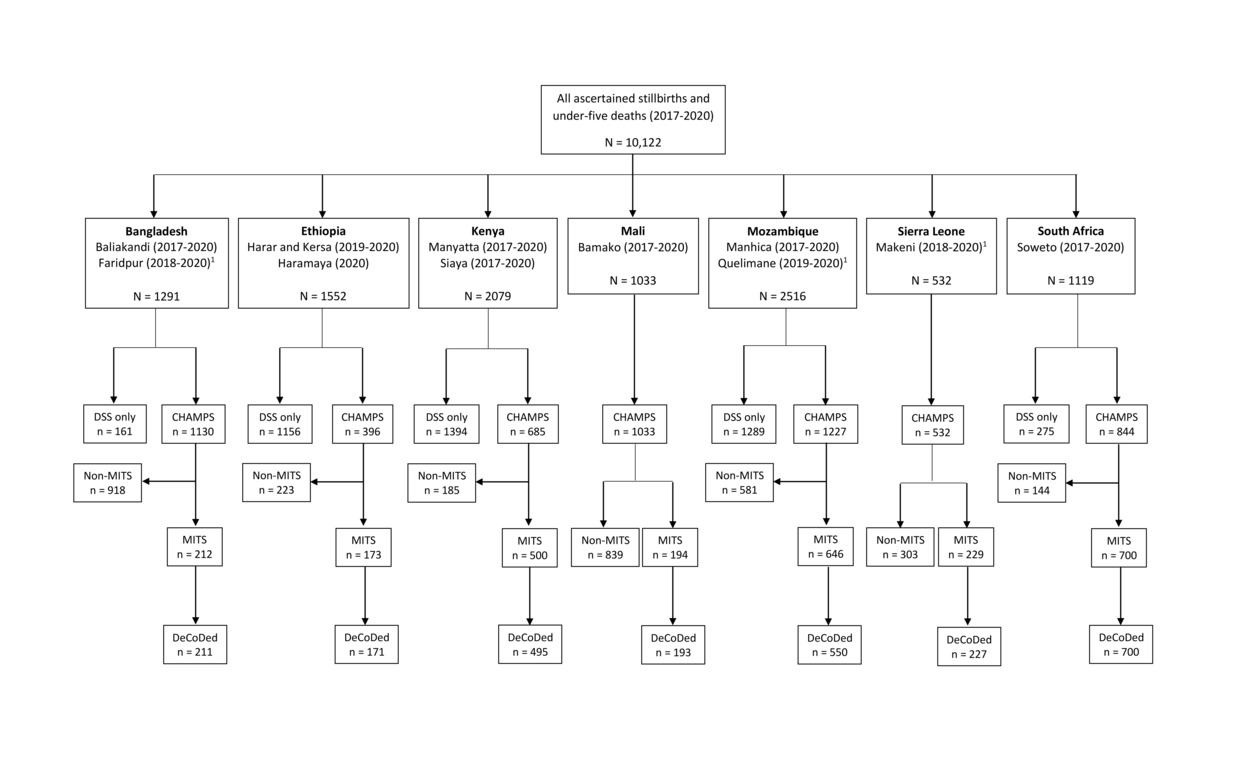

Supplement: Supplementary file 1 — Figure S1: Flow diagram from ascertainment to CHAMPS enrolment, MITS performed and cause of death determination, by site. DSS only refers to deaths captured in the DSS but never enrolled in CHAMPS; non‐MITS refers to deaths enrolled in CHAMPS but for whom MITS was not performed; MITS refers to deaths enrolled in CHAMPS and for whom MITS was performed; DeCoDed refers to deaths for whom MITS was performed and reviewed by the DeCoDe panel as of 24 May 2022. All‐cause age‐specific mortality rates from the DHS were substituted during calculations for catchments without DSS data availability. CHAMPS, Child Health and Mortality Prevention Surveillance Network; children (1–5 years), DeCoDe, determination of cause of death; DHS, Demographic and Health Surveys Program; DSS, demographic surveillance system; infants (29–365 days); MITS, minimally invasive tissue sampling; neonates, neonates (0–28 days); stillbirths (no spontaneous breathing or movement at time of delivery and [1] weighing > 1 kg and/or [2] estimated gestational age ≥ 28 weeks). Figure S2: Venn diagrams of enrolment and MITS performed among all ascertained deaths in the CHAMPS Network, by site and age. In sites with available DSS data, it is assumed all CHAMPS cases are also captured in the DSS system. (1) DSS data are ignored due to discordant availability among catchments. (2) DSS data included in count of non‐MITS CHAMPS cases. (3) DSS data are not available. (4) Stillbirths (no spontaneous breathing or movement at time of delivery and [1] weighing > 1 kg and/or [2] estimated gestational age ≥ 28 weeks); neonates (0–28 days); infants (29–365 days); children (1–5 years). (5) Combined for all catchments with available DSS data. CHAMPS, Child Health and Mortality Prevention Surveillance Network; DSS, demographic surveillance system; MITS, minimally invasive tissue sampling. Figure S3: Fractions for the most frequent perinatal and paediatric causes of death in the CHAMPS Network, 2017–2020. (A) Stillbirths, no sp [file PPE-39-698-s001.zip › ppe70067-sup-0001-FigureS1@Suppl Figure 1. Flow Diagram.jpg]

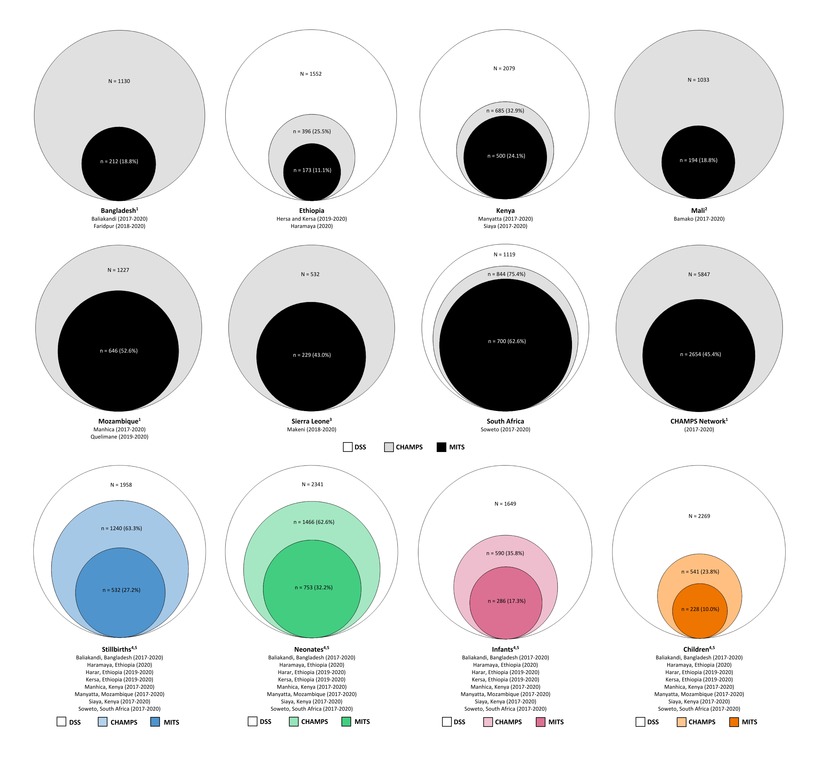

Supplement: Supplementary file 1 — Figure S1: Flow diagram from ascertainment to CHAMPS enrolment, MITS performed and cause of death determination, by site. DSS only refers to deaths captured in the DSS but never enrolled in CHAMPS; non‐MITS refers to deaths enrolled in CHAMPS but for whom MITS was not performed; MITS refers to deaths enrolled in CHAMPS and for whom MITS was performed; DeCoDed refers to deaths for whom MITS was performed and reviewed by the DeCoDe panel as of 24 May 2022. All‐cause age‐specific mortality rates from the DHS were substituted during calculations for catchments without DSS data availability. CHAMPS, Child Health and Mortality Prevention Surveillance Network; children (1–5 years), DeCoDe, determination of cause of death; DHS, Demographic and Health Surveys Program; DSS, demographic surveillance system; infants (29–365 days); MITS, minimally invasive tissue sampling; neonates, neonates (0–28 days); stillbirths (no spontaneous breathing or movement at time of delivery and [1] weighing > 1 kg and/or [2] estimated gestational age ≥ 28 weeks). Figure S2: Venn diagrams of enrolment and MITS performed among all ascertained deaths in the CHAMPS Network, by site and age. In sites with available DSS data, it is assumed all CHAMPS cases are also captured in the DSS system. (1) DSS data are ignored due to discordant availability among catchments. (2) DSS data included in count of non‐MITS CHAMPS cases. (3) DSS data are not available. (4) Stillbirths (no spontaneous breathing or movement at time of delivery and [1] weighing > 1 kg and/or [2] estimated gestational age ≥ 28 weeks); neonates (0–28 days); infants (29–365 days); children (1–5 years). (5) Combined for all catchments with available DSS data. CHAMPS, Child Health and Mortality Prevention Surveillance Network; DSS, demographic surveillance system; MITS, minimally invasive tissue sampling. Figure S3: Fractions for the most frequent perinatal and paediatric causes of death in the CHAMPS Network, 2017–2020. (A) Stillbirths, no sp [file PPE-39-698-s001.zip › ppe70067-sup-0002-FigureS2@Suppl Figure 2. Venn Diagrams.jpg]

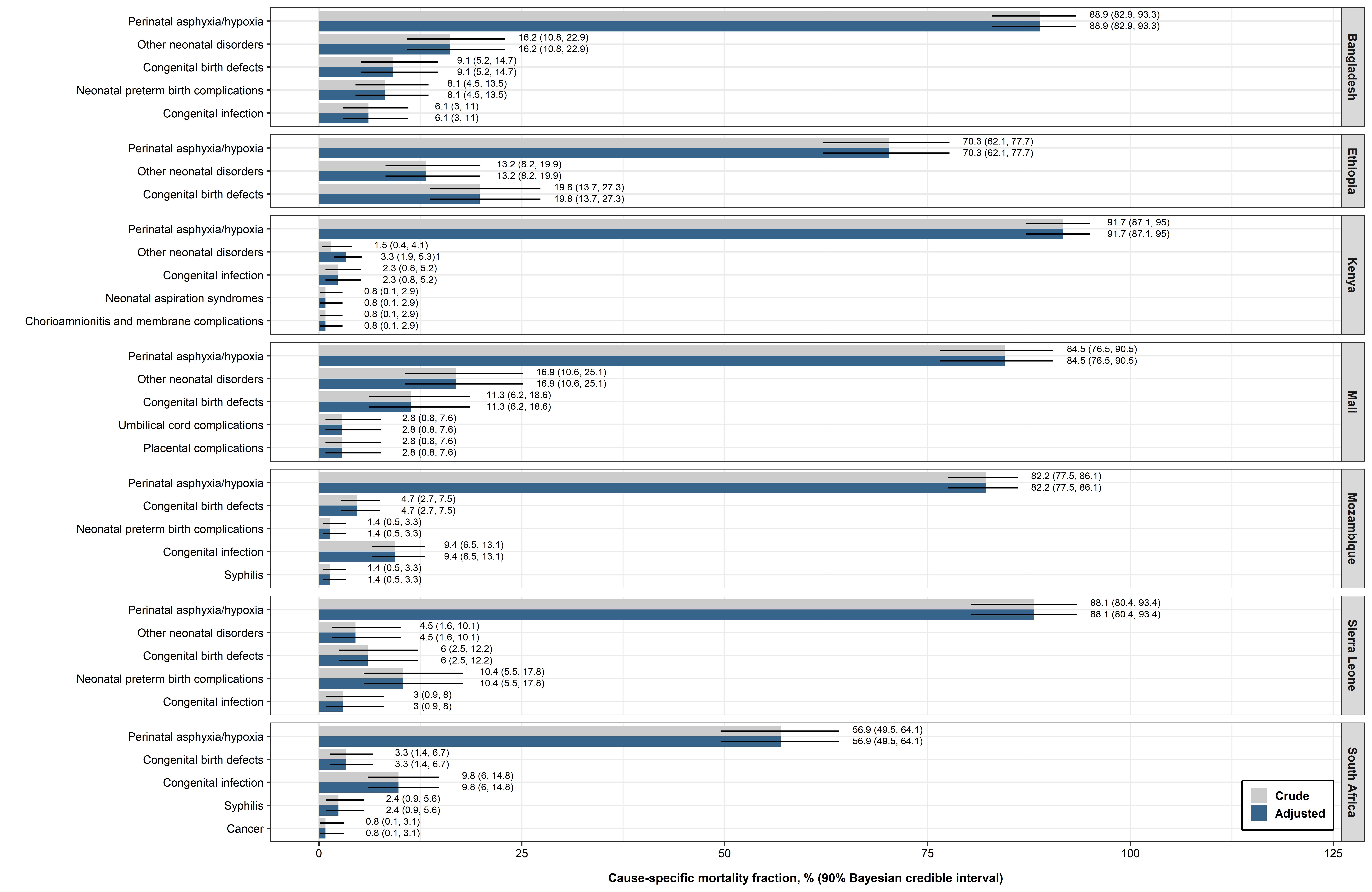

Supplement: Supplementary file 1 — Figure S1: Flow diagram from ascertainment to CHAMPS enrolment, MITS performed and cause of death determination, by site. DSS only refers to deaths captured in the DSS but never enrolled in CHAMPS; non‐MITS refers to deaths enrolled in CHAMPS but for whom MITS was not performed; MITS refers to deaths enrolled in CHAMPS and for whom MITS was performed; DeCoDed refers to deaths for whom MITS was performed and reviewed by the DeCoDe panel as of 24 May 2022. All‐cause age‐specific mortality rates from the DHS were substituted during calculations for catchments without DSS data availability. CHAMPS, Child Health and Mortality Prevention Surveillance Network; children (1–5 years), DeCoDe, determination of cause of death; DHS, Demographic and Health Surveys Program; DSS, demographic surveillance system; infants (29–365 days); MITS, minimally invasive tissue sampling; neonates, neonates (0–28 days); stillbirths (no spontaneous breathing or movement at time of delivery and [1] weighing > 1 kg and/or [2] estimated gestational age ≥ 28 weeks). Figure S2: Venn diagrams of enrolment and MITS performed among all ascertained deaths in the CHAMPS Network, by site and age. In sites with available DSS data, it is assumed all CHAMPS cases are also captured in the DSS system. (1) DSS data are ignored due to discordant availability among catchments. (2) DSS data included in count of non‐MITS CHAMPS cases. (3) DSS data are not available. (4) Stillbirths (no spontaneous breathing or movement at time of delivery and [1] weighing > 1 kg and/or [2] estimated gestational age ≥ 28 weeks); neonates (0–28 days); infants (29–365 days); children (1–5 years). (5) Combined for all catchments with available DSS data. CHAMPS, Child Health and Mortality Prevention Surveillance Network; DSS, demographic surveillance system; MITS, minimally invasive tissue sampling. Figure S3: Fractions for the most frequent perinatal and paediatric causes of death in the CHAMPS Network, 2017–2020. (A) Stillbirths, no sp [file PPE-39-698-s001.zip › ppe70067-sup-0003-FigureS3@Suppl Figure 3A. Stillbirths.jpg]

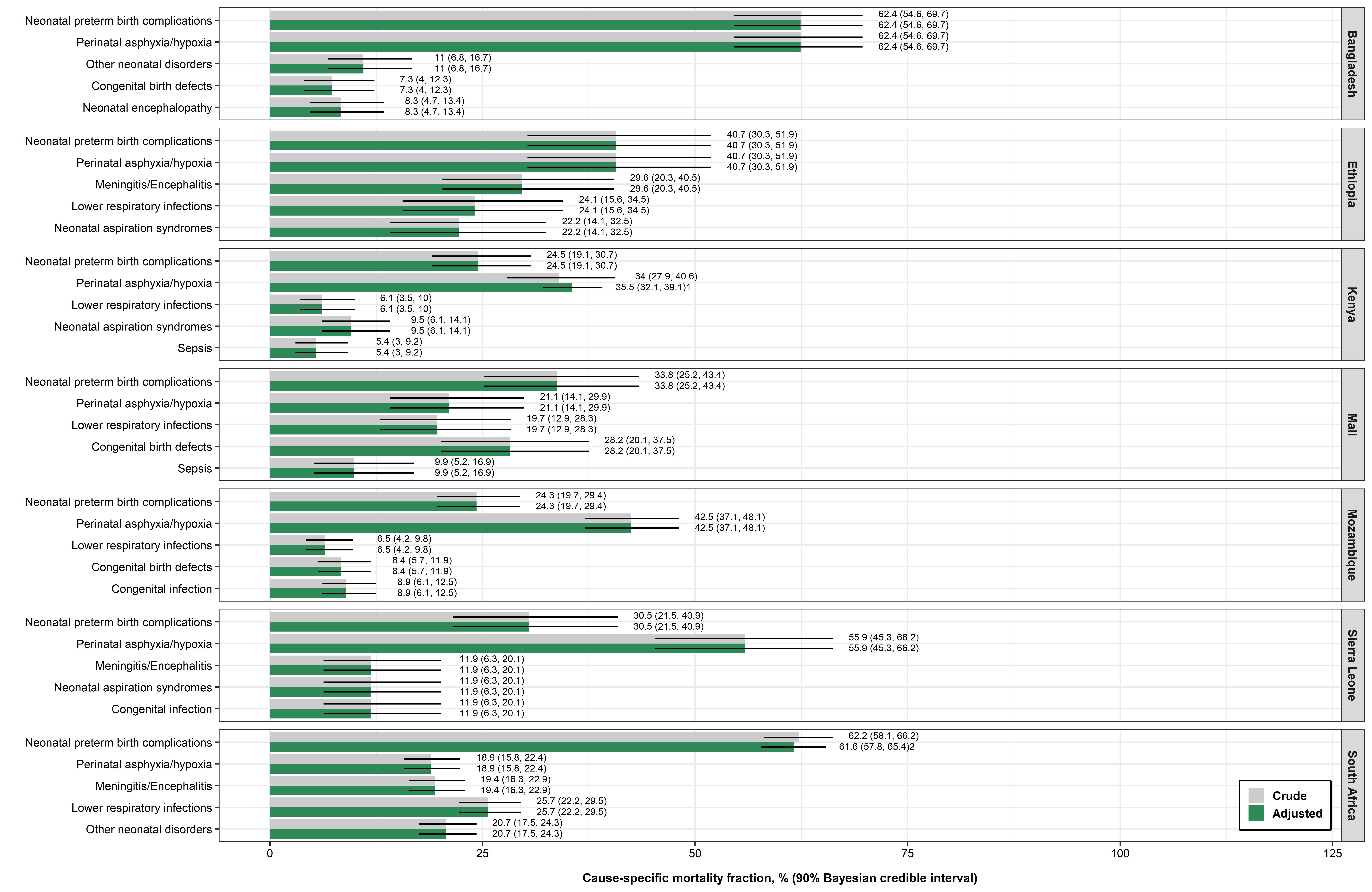

Supplement: Supplementary file 1 — Figure S1: Flow diagram from ascertainment to CHAMPS enrolment, MITS performed and cause of death determination, by site. DSS only refers to deaths captured in the DSS but never enrolled in CHAMPS; non‐MITS refers to deaths enrolled in CHAMPS but for whom MITS was not performed; MITS refers to deaths enrolled in CHAMPS and for whom MITS was performed; DeCoDed refers to deaths for whom MITS was performed and reviewed by the DeCoDe panel as of 24 May 2022. All‐cause age‐specific mortality rates from the DHS were substituted during calculations for catchments without DSS data availability. CHAMPS, Child Health and Mortality Prevention Surveillance Network; children (1–5 years), DeCoDe, determination of cause of death; DHS, Demographic and Health Surveys Program; DSS, demographic surveillance system; infants (29–365 days); MITS, minimally invasive tissue sampling; neonates, neonates (0–28 days); stillbirths (no spontaneous breathing or movement at time of delivery and [1] weighing > 1 kg and/or [2] estimated gestational age ≥ 28 weeks). Figure S2: Venn diagrams of enrolment and MITS performed among all ascertained deaths in the CHAMPS Network, by site and age. In sites with available DSS data, it is assumed all CHAMPS cases are also captured in the DSS system. (1) DSS data are ignored due to discordant availability among catchments. (2) DSS data included in count of non‐MITS CHAMPS cases. (3) DSS data are not available. (4) Stillbirths (no spontaneous breathing or movement at time of delivery and [1] weighing > 1 kg and/or [2] estimated gestational age ≥ 28 weeks); neonates (0–28 days); infants (29–365 days); children (1–5 years). (5) Combined for all catchments with available DSS data. CHAMPS, Child Health and Mortality Prevention Surveillance Network; DSS, demographic surveillance system; MITS, minimally invasive tissue sampling. Figure S3: Fractions for the most frequent perinatal and paediatric causes of death in the CHAMPS Network, 2017–2020. (A) Stillbirths, no sp [file PPE-39-698-s001.zip › ppe70067-sup-0004-FigureS3@Suppl Figure 3B. Neonates.jpg]

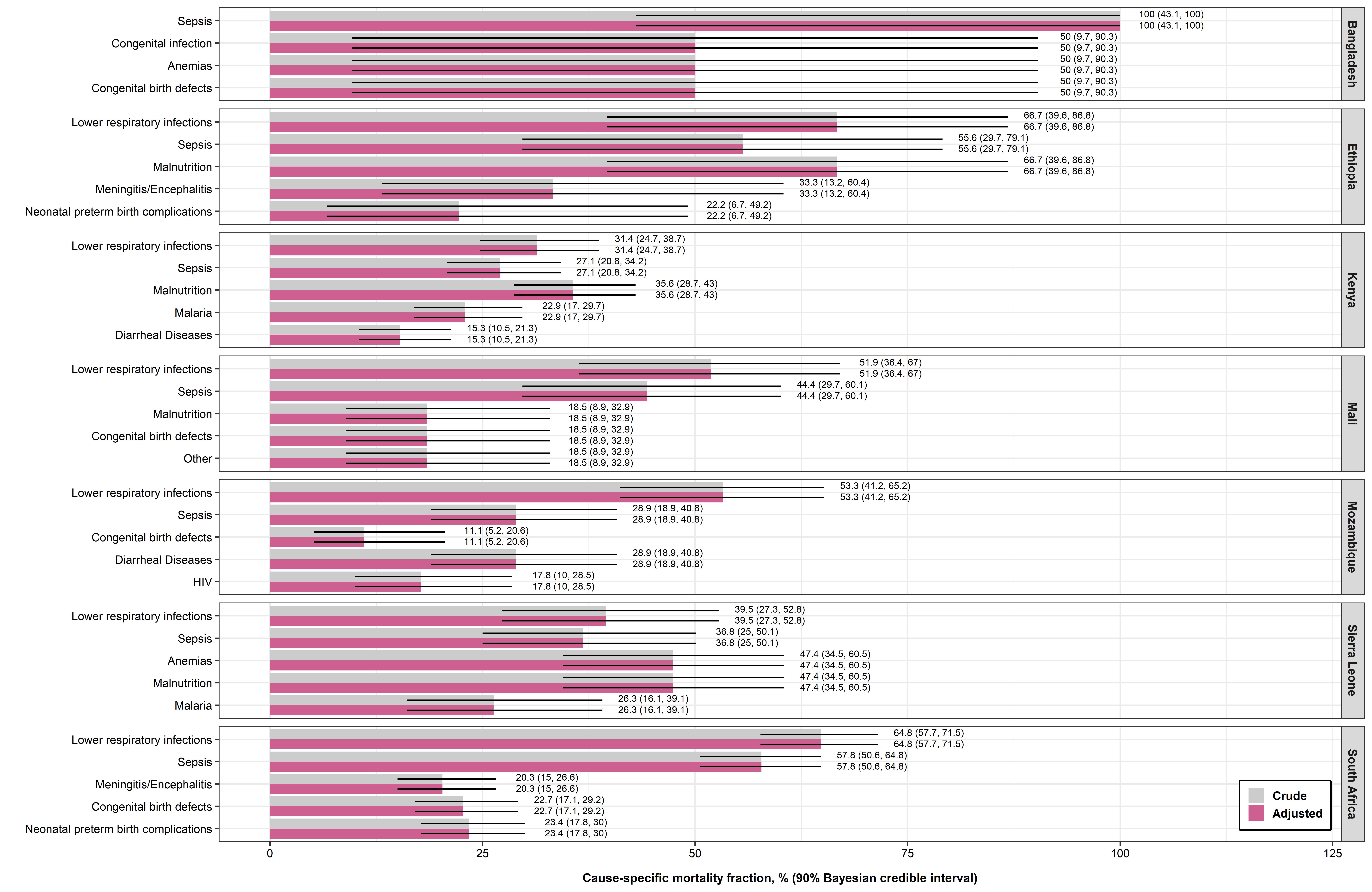

Supplement: Supplementary file 1 — Figure S1: Flow diagram from ascertainment to CHAMPS enrolment, MITS performed and cause of death determination, by site. DSS only refers to deaths captured in the DSS but never enrolled in CHAMPS; non‐MITS refers to deaths enrolled in CHAMPS but for whom MITS was not performed; MITS refers to deaths enrolled in CHAMPS and for whom MITS was performed; DeCoDed refers to deaths for whom MITS was performed and reviewed by the DeCoDe panel as of 24 May 2022. All‐cause age‐specific mortality rates from the DHS were substituted during calculations for catchments without DSS data availability. CHAMPS, Child Health and Mortality Prevention Surveillance Network; children (1–5 years), DeCoDe, determination of cause of death; DHS, Demographic and Health Surveys Program; DSS, demographic surveillance system; infants (29–365 days); MITS, minimally invasive tissue sampling; neonates, neonates (0–28 days); stillbirths (no spontaneous breathing or movement at time of delivery and [1] weighing > 1 kg and/or [2] estimated gestational age ≥ 28 weeks). Figure S2: Venn diagrams of enrolment and MITS performed among all ascertained deaths in the CHAMPS Network, by site and age. In sites with available DSS data, it is assumed all CHAMPS cases are also captured in the DSS system. (1) DSS data are ignored due to discordant availability among catchments. (2) DSS data included in count of non‐MITS CHAMPS cases. (3) DSS data are not available. (4) Stillbirths (no spontaneous breathing or movement at time of delivery and [1] weighing > 1 kg and/or [2] estimated gestational age ≥ 28 weeks); neonates (0–28 days); infants (29–365 days); children (1–5 years). (5) Combined for all catchments with available DSS data. CHAMPS, Child Health and Mortality Prevention Surveillance Network; DSS, demographic surveillance system; MITS, minimally invasive tissue sampling. Figure S3: Fractions for the most frequent perinatal and paediatric causes of death in the CHAMPS Network, 2017–2020. (A) Stillbirths, no sp [file PPE-39-698-s001.zip › ppe70067-sup-0005-FigureS3@Suppl Figure 3C. Infants.jpg]

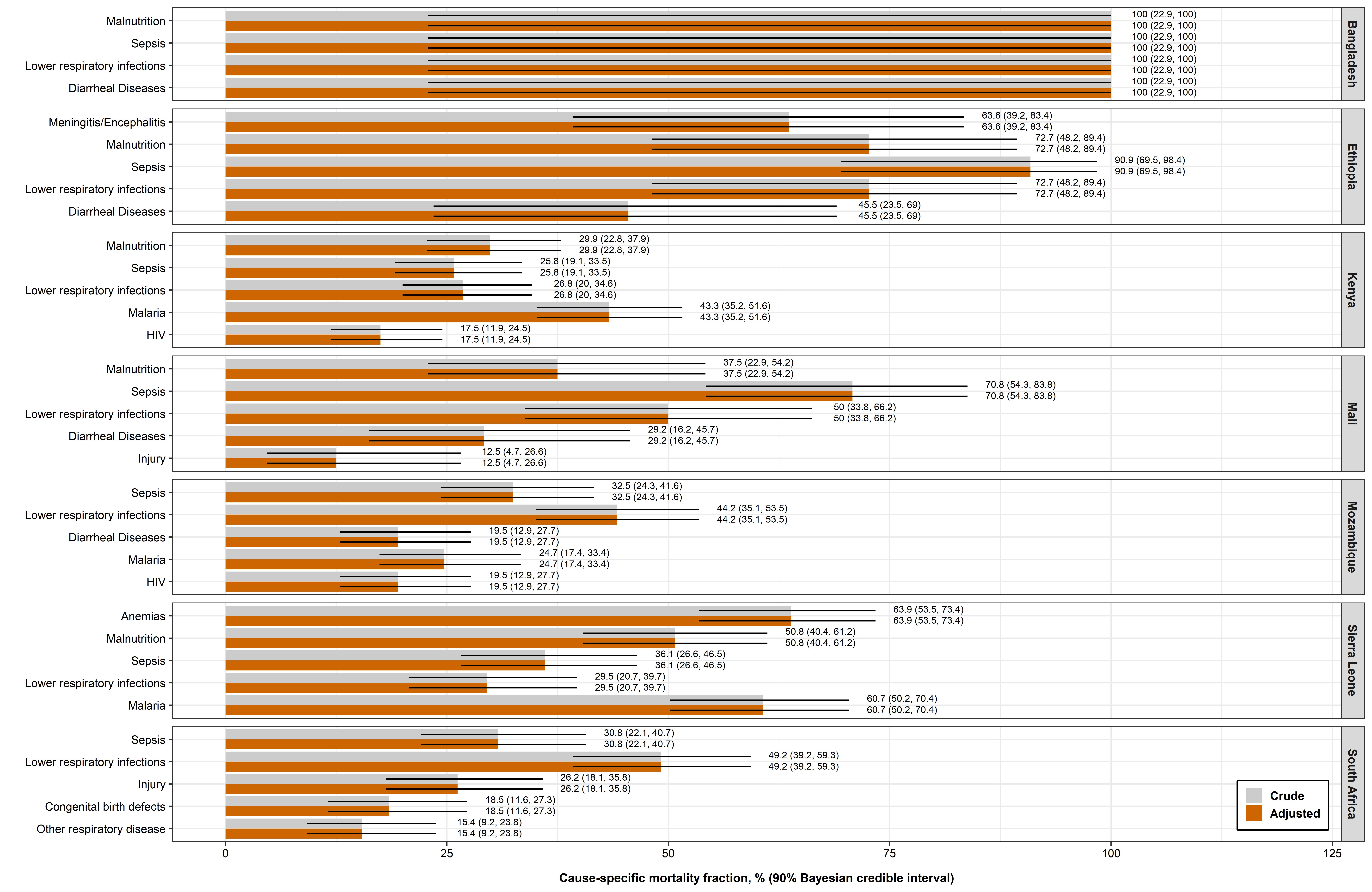

Supplement: Supplementary file 1 — Figure S1: Flow diagram from ascertainment to CHAMPS enrolment, MITS performed and cause of death determination, by site. DSS only refers to deaths captured in the DSS but never enrolled in CHAMPS; non‐MITS refers to deaths enrolled in CHAMPS but for whom MITS was not performed; MITS refers to deaths enrolled in CHAMPS and for whom MITS was performed; DeCoDed refers to deaths for whom MITS was performed and reviewed by the DeCoDe panel as of 24 May 2022. All‐cause age‐specific mortality rates from the DHS were substituted during calculations for catchments without DSS data availability. CHAMPS, Child Health and Mortality Prevention Surveillance Network; children (1–5 years), DeCoDe, determination of cause of death; DHS, Demographic and Health Surveys Program; DSS, demographic surveillance system; infants (29–365 days); MITS, minimally invasive tissue sampling; neonates, neonates (0–28 days); stillbirths (no spontaneous breathing or movement at time of delivery and [1] weighing > 1 kg and/or [2] estimated gestational age ≥ 28 weeks). Figure S2: Venn diagrams of enrolment and MITS performed among all ascertained deaths in the CHAMPS Network, by site and age. In sites with available DSS data, it is assumed all CHAMPS cases are also captured in the DSS system. (1) DSS data are ignored due to discordant availability among catchments. (2) DSS data included in count of non‐MITS CHAMPS cases. (3) DSS data are not available. (4) Stillbirths (no spontaneous breathing or movement at time of delivery and [1] weighing > 1 kg and/or [2] estimated gestational age ≥ 28 weeks); neonates (0–28 days); infants (29–365 days); children (1–5 years). (5) Combined for all catchments with available DSS data. CHAMPS, Child Health and Mortality Prevention Surveillance Network; DSS, demographic surveillance system; MITS, minimally invasive tissue sampling. Figure S3: Fractions for the most frequent perinatal and paediatric causes of death in the CHAMPS Network, 2017–2020. (A) Stillbirths, no sp [file PPE-39-698-s001.zip › ppe70067-sup-0006-FigureS3@Suppl Figure 3D. Children.jpg]

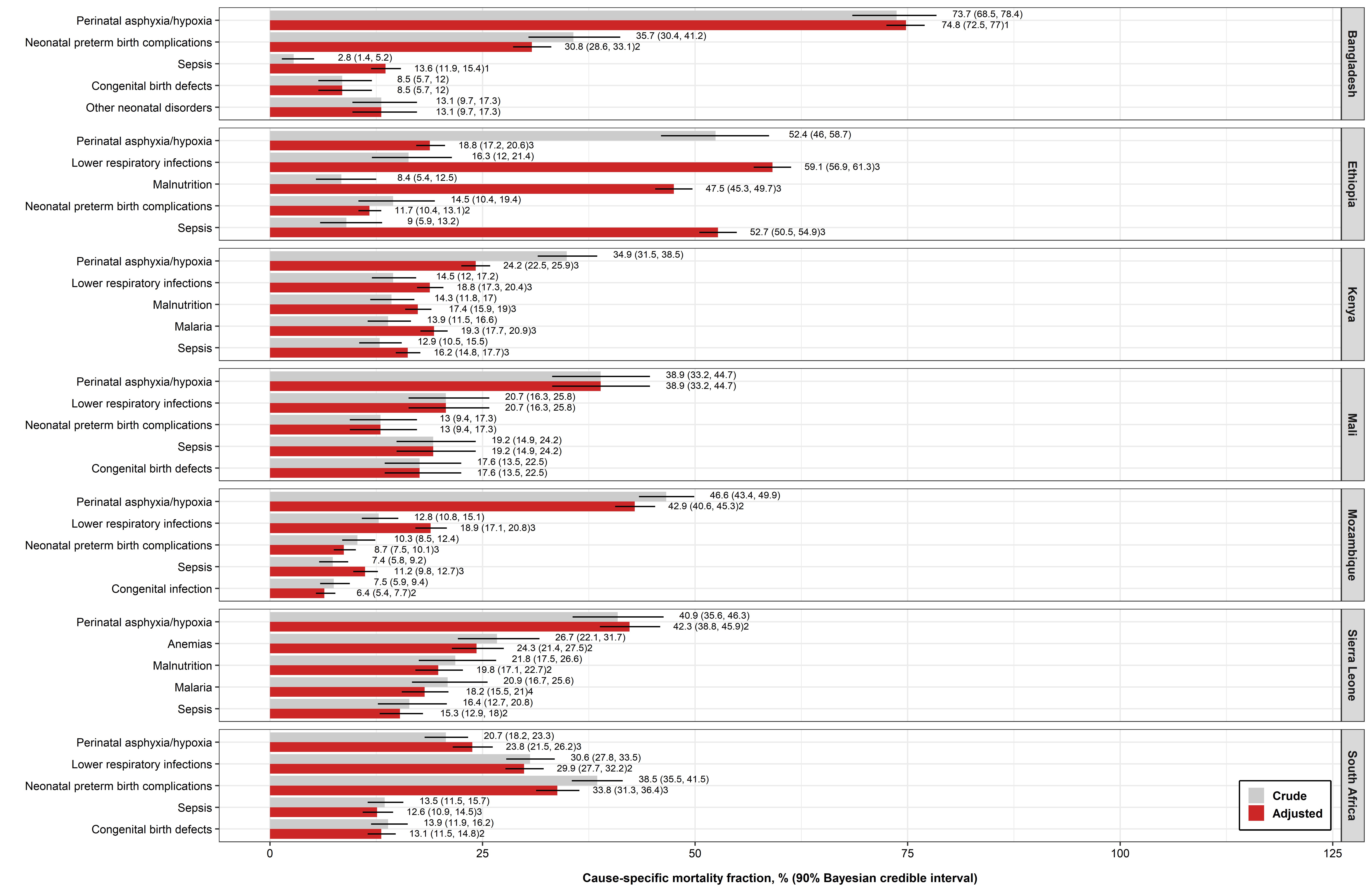

Supplement: Supplementary file 1 — Figure S1: Flow diagram from ascertainment to CHAMPS enrolment, MITS performed and cause of death determination, by site. DSS only refers to deaths captured in the DSS but never enrolled in CHAMPS; non‐MITS refers to deaths enrolled in CHAMPS but for whom MITS was not performed; MITS refers to deaths enrolled in CHAMPS and for whom MITS was performed; DeCoDed refers to deaths for whom MITS was performed and reviewed by the DeCoDe panel as of 24 May 2022. All‐cause age‐specific mortality rates from the DHS were substituted during calculations for catchments without DSS data availability. CHAMPS, Child Health and Mortality Prevention Surveillance Network; children (1–5 years), DeCoDe, determination of cause of death; DHS, Demographic and Health Surveys Program; DSS, demographic surveillance system; infants (29–365 days); MITS, minimally invasive tissue sampling; neonates, neonates (0–28 days); stillbirths (no spontaneous breathing or movement at time of delivery and [1] weighing > 1 kg and/or [2] estimated gestational age ≥ 28 weeks). Figure S2: Venn diagrams of enrolment and MITS performed among all ascertained deaths in the CHAMPS Network, by site and age. In sites with available DSS data, it is assumed all CHAMPS cases are also captured in the DSS system. (1) DSS data are ignored due to discordant availability among catchments. (2) DSS data included in count of non‐MITS CHAMPS cases. (3) DSS data are not available. (4) Stillbirths (no spontaneous breathing or movement at time of delivery and [1] weighing > 1 kg and/or [2] estimated gestational age ≥ 28 weeks); neonates (0–28 days); infants (29–365 days); children (1–5 years). (5) Combined for all catchments with available DSS data. CHAMPS, Child Health and Mortality Prevention Surveillance Network; DSS, demographic surveillance system; MITS, minimally invasive tissue sampling. Figure S3: Fractions for the most frequent perinatal and paediatric causes of death in the CHAMPS Network, 2017–2020. (A) Stillbirths, no sp [file PPE-39-698-s001.zip › ppe70067-sup-0007-FigureS3@Suppl Figure 3E. Under-Five.jpg]

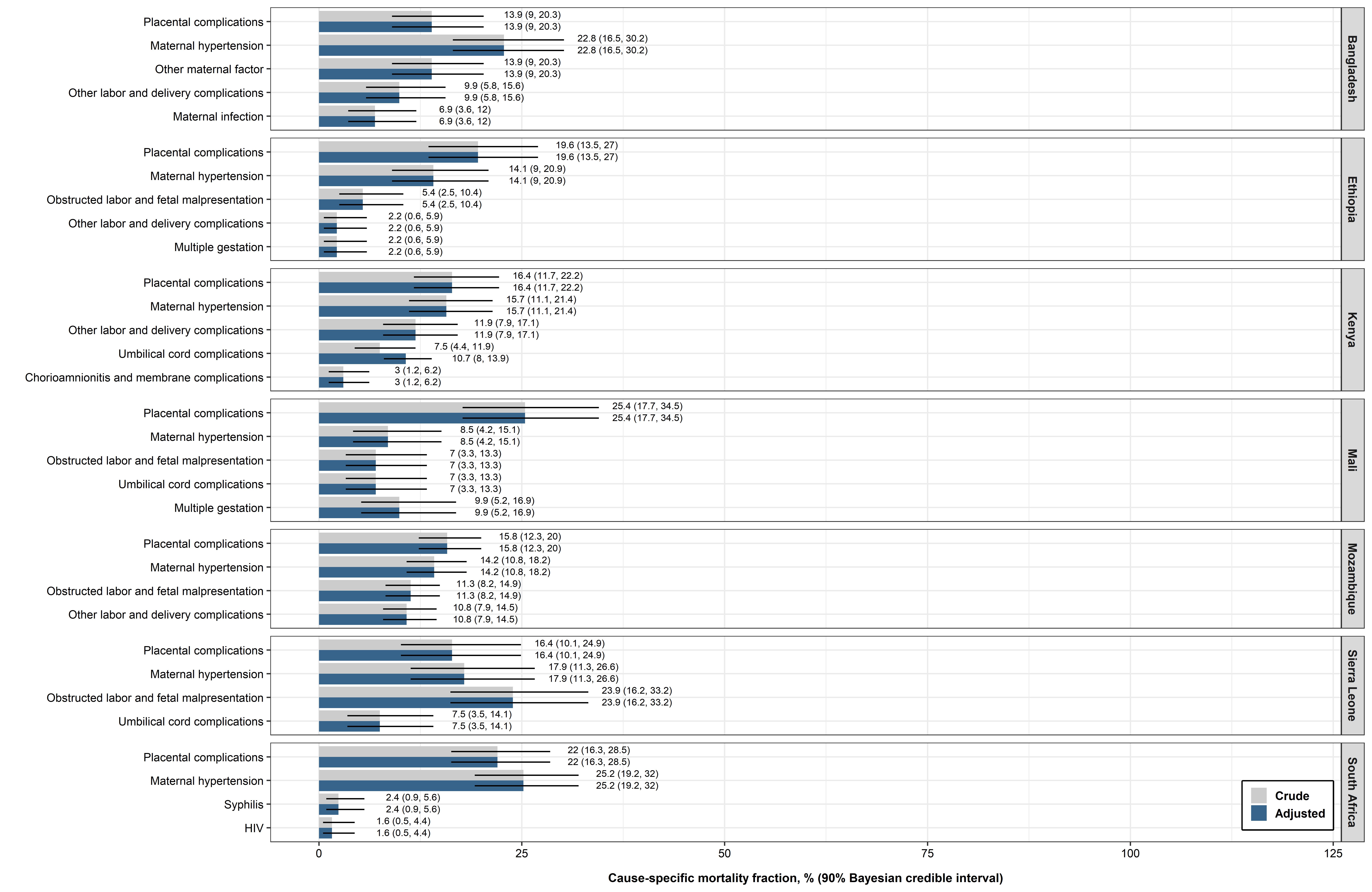

Supplement: Supplementary file 1 — Figure S1: Flow diagram from ascertainment to CHAMPS enrolment, MITS performed and cause of death determination, by site. DSS only refers to deaths captured in the DSS but never enrolled in CHAMPS; non‐MITS refers to deaths enrolled in CHAMPS but for whom MITS was not performed; MITS refers to deaths enrolled in CHAMPS and for whom MITS was performed; DeCoDed refers to deaths for whom MITS was performed and reviewed by the DeCoDe panel as of 24 May 2022. All‐cause age‐specific mortality rates from the DHS were substituted during calculations for catchments without DSS data availability. CHAMPS, Child Health and Mortality Prevention Surveillance Network; children (1–5 years), DeCoDe, determination of cause of death; DHS, Demographic and Health Surveys Program; DSS, demographic surveillance system; infants (29–365 days); MITS, minimally invasive tissue sampling; neonates, neonates (0–28 days); stillbirths (no spontaneous breathing or movement at time of delivery and [1] weighing > 1 kg and/or [2] estimated gestational age ≥ 28 weeks). Figure S2: Venn diagrams of enrolment and MITS performed among all ascertained deaths in the CHAMPS Network, by site and age. In sites with available DSS data, it is assumed all CHAMPS cases are also captured in the DSS system. (1) DSS data are ignored due to discordant availability among catchments. (2) DSS data included in count of non‐MITS CHAMPS cases. (3) DSS data are not available. (4) Stillbirths (no spontaneous breathing or movement at time of delivery and [1] weighing > 1 kg and/or [2] estimated gestational age ≥ 28 weeks); neonates (0–28 days); infants (29–365 days); children (1–5 years). (5) Combined for all catchments with available DSS data. CHAMPS, Child Health and Mortality Prevention Surveillance Network; DSS, demographic surveillance system; MITS, minimally invasive tissue sampling. Figure S3: Fractions for the most frequent perinatal and paediatric causes of death in the CHAMPS Network, 2017–2020. (A) Stillbirths, no sp [file PPE-39-698-s001.zip › ppe70067-sup-0008-FigureS4@Suppl Figure 4A. Stillbirths.jpg]

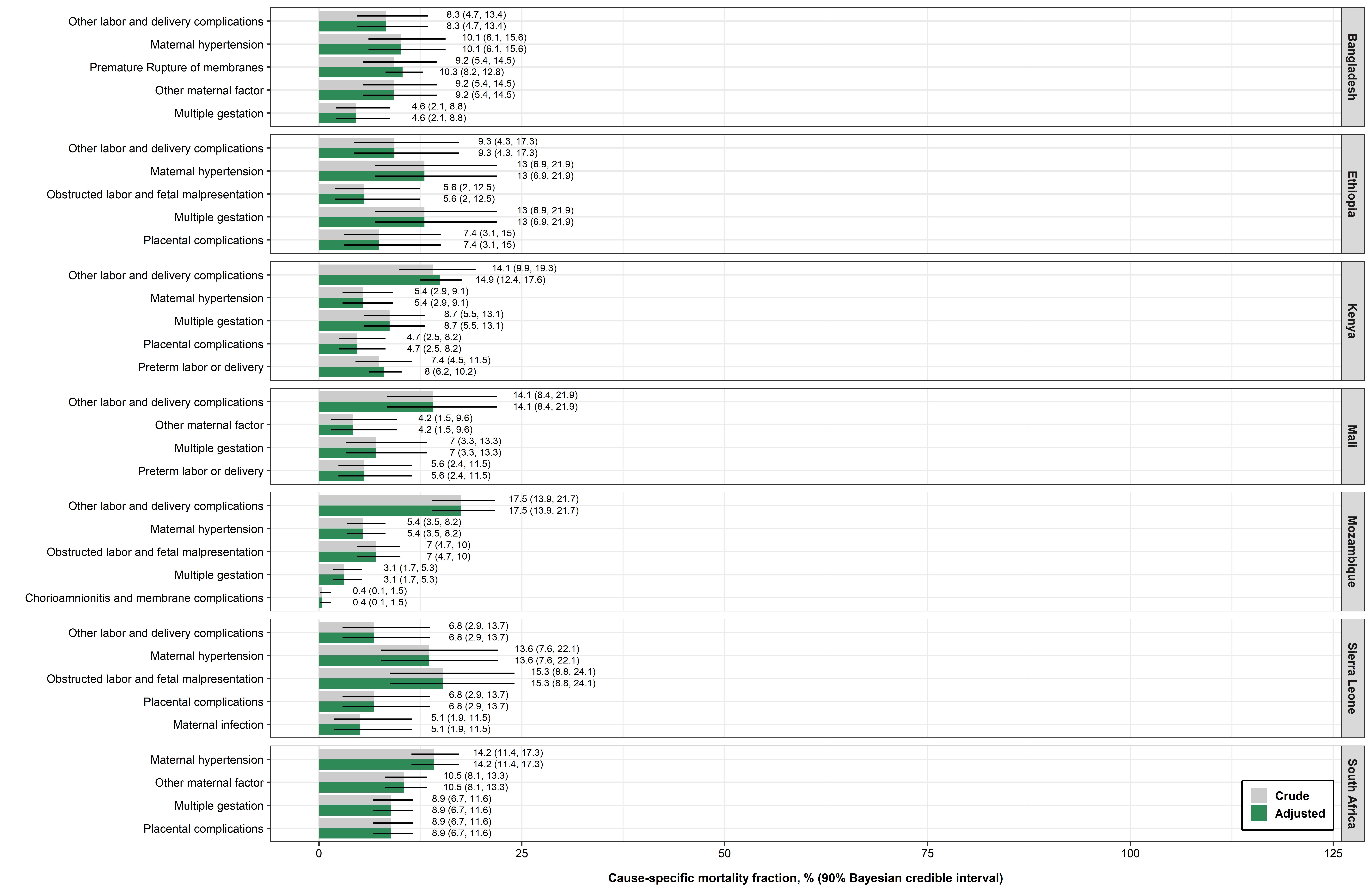

Supplement: Supplementary file 1 — Figure S1: Flow diagram from ascertainment to CHAMPS enrolment, MITS performed and cause of death determination, by site. DSS only refers to deaths captured in the DSS but never enrolled in CHAMPS; non‐MITS refers to deaths enrolled in CHAMPS but for whom MITS was not performed; MITS refers to deaths enrolled in CHAMPS and for whom MITS was performed; DeCoDed refers to deaths for whom MITS was performed and reviewed by the DeCoDe panel as of 24 May 2022. All‐cause age‐specific mortality rates from the DHS were substituted during calculations for catchments without DSS data availability. CHAMPS, Child Health and Mortality Prevention Surveillance Network; children (1–5 years), DeCoDe, determination of cause of death; DHS, Demographic and Health Surveys Program; DSS, demographic surveillance system; infants (29–365 days); MITS, minimally invasive tissue sampling; neonates, neonates (0–28 days); stillbirths (no spontaneous breathing or movement at time of delivery and [1] weighing > 1 kg and/or [2] estimated gestational age ≥ 28 weeks). Figure S2: Venn diagrams of enrolment and MITS performed among all ascertained deaths in the CHAMPS Network, by site and age. In sites with available DSS data, it is assumed all CHAMPS cases are also captured in the DSS system. (1) DSS data are ignored due to discordant availability among catchments. (2) DSS data included in count of non‐MITS CHAMPS cases. (3) DSS data are not available. (4) Stillbirths (no spontaneous breathing or movement at time of delivery and [1] weighing > 1 kg and/or [2] estimated gestational age ≥ 28 weeks); neonates (0–28 days); infants (29–365 days); children (1–5 years). (5) Combined for all catchments with available DSS data. CHAMPS, Child Health and Mortality Prevention Surveillance Network; DSS, demographic surveillance system; MITS, minimally invasive tissue sampling. Figure S3: Fractions for the most frequent perinatal and paediatric causes of death in the CHAMPS Network, 2017–2020. (A) Stillbirths, no sp [file PPE-39-698-s001.zip › ppe70067-sup-0009-FigureS4@Suppl Figure 4B. Neonates.jpg]
